# A Bioinspired Insole Based on Shear-Stiffening Gel for Plantar Load Modulation: Material Characterization and a Single-Case Wearable Evaluation

**DOI:** 10.3390/biomimetics11100699

**Published:** 2026-10-02

**Authors:** Qingqing Zhang, Jiaming Chen, Changqiu Zhou, Ziqin Ling, Kehan Zou, Wenbo Yuan, Jiangcheng Chen, Ning Xi

**Affiliations:** Department of Data and Systems Engineering, The University of Hong Kong, Hong Kong 999077, China; u3008422@connect.hku.hk (J.C.); zhoucq@connect.hku.hk (C.Z.); zqling@connect.hku.hk (Z.L.); u3008454@connect.hku.hk (K.Z.); ywb2022@connect.hku.hk (W.Y.); jcchen@hku.hk (J.C.)

**Keywords:** bioinspired insole, shear-stiffening gels, adaptive stiffness, plantar loading, plantar mechanics, gait biomechanics

## Abstract

The human foot adaptively modulates stiffness during walking to balance impact attenuation, arch support, and propulsion, but this function may decline with aging and foot deformity. This study developed a bioinspired insole (bio-insole) for passive rate-adaptive plantar load modulation by embedding shear-stiffening gel (STG) within a Dragon Skin 30 (DS30) silicone elastomer backbone. DS30-STG sandwich specimens were characterized under rate-dependent and cyclic compression. A customized prototype was then evaluated in a preliminary single-case proof-of-concept walking test involving an older adult with sarcopenia, pes cavus, and hindfoot varus tendency, using stock and rigid insoles as comparators. The DS30-STG composite exhibited rate-dependent stiffening, with the apparent compressive modulus increasing from 262.11 to 553.00 kPa as the compression speed increased from 30 to 2000 mm/min, and showed short-term repeatable responses over 100 cycles at 40% strain. Compared with the stock insole, the bio-insole was associated with higher pressure-derived plantar force and peak pressure at the heel, medial arch, and first metatarsophalangeal joint. It also showed lower measured peak electromyography amplitudes of the tibialis anterior and lateral gastrocnemius by 7.8% and 9.3%, respectively, compared with the stock insole. No large observable changes were found in sagittal-plane ankle or knee excursion. These preliminary participant-specific findings suggest that the tested DS30-STG-based insole was associated with altered regional plantar loading and lower-leg muscle activation patterns during walking. Controlled studies with larger cohorts are required to determine reproducibility, tissue-loading safety, functional benefit, and clinical relevance.

## 1. Introduction

The human foot is the body’s primary mechanical interface with the ground and plays a central role in load bearing, balance regulation, shock attenuation, and forward propulsion during walking [1,2]. Rather than having fixed mechanical properties, the foot functions as an adaptive biomechanical system whose effective stiffness changes across the gait cycle. During initial contact, the foot–ankle complex remains relatively compliant to attenuate impact and accommodate the ground surface [3]. During midstance, the medial longitudinal arch and surrounding soft tissues support load-bearing stability. In late stance and push-off, the foot becomes functionally stiffer, forming a rigid lever that enhances the transmission of ankle-generated power and facilitates propulsion [4]. This transition between compliance and rigidity is regulated by the coordinated action of intrinsic foot muscles, ligaments, and the plantar fascia, particularly through the windlass mechanism [5,6,7,8]. Such gait-phase-dependent stiffness modulation enables the foot to balance adaptability, stability, and mechanical efficiency.

This adaptive capacity can be compromised by aging and musculoskeletal disorders. Sarcopenia, reduced soft-tissue elasticity, degenerative changes in ligaments and tendons, impaired proprioception, and diminished neuromuscular coordination may impair foot stiffness regulation and plantar load control during walking [9,10,11,12,13,14,15,16]. Structural deformities, including pes cavus, excessive pronation, and hindfoot varus, can further alter plantar contact and redistribute loading away from mechanically favorable regions [11,17,18,19,20,21]. Pes cavus, characterized by an elevated medial longitudinal arch and reduced midfoot contact, often increases loading under the heel and forefoot and alters center-of-pressure progression [21,22]. Hindfoot varus may additionally promote lateral border loading, reduce rearfoot stability, and increase the demand for active ankle control [23]. These effects may be particularly problematic in older adults with sarcopenia, in whom reduced muscle strength and power are associated with impaired mobility and increased fall risk [24,25]. Therefore, footwear interfaces that can assist plantar load management in aging or pathological feet remain an important design objective. In the present study, fall risk is cited as part of the broader clinical rationale for improving plantar-support design in mechanically compromised older feet, rather than as an outcome directly assessed in the wearable evaluation.

Insoles are widely used to improve comfort, redistribute plantar pressure, and provide biomechanical support for individuals with gait abnormalities or foot disorders. Soft insoles can enhance cushioning and reduce local pressure concentrations, but they may provide limited structural support during arch loading and forefoot load transfer [26,27]. In contrast, rigid or semi-rigid insoles, including carbon-fiber-based inserts, can increase footwear bending stiffness, improve forefoot lever function, and influence plantar pressure distribution during push-off [28,29,30,31]. More recent approaches, such as customized, digitally optimized, and 3D-printed insoles, have been developed to redistribute plantar pressure according to individual foot morphology or pathological loading patterns, particularly in diabetic or high-risk feet [32,33,34]. Footwear studies have also shown that tuning longitudinal bending stiffness or energy-return properties can affect ankle–foot mechanics, propulsion, and locomotor energetics [35,36]. Despite these advances, most existing insoles provide predetermined mechanical properties after fabrication, with limited capacity for passive, localized adaptation to loading rate, gait phase, or regional plantar demand.

Variable-stiffness ankle–foot prostheses and wearable gait-assistance devices demonstrate that modulating ankle–foot impedance can improve locomotor assistance [37,38,39]. However, these systems typically rely on springs, clutches, actuators, or control mechanisms and primarily provide joint-level assistance rather than localized plantar-region support. This limitation motivates the development of compact, passive, and material-based insoles that can adapt their mechanical response to changing plantar-loading conditions. An insole that remains compliant under slow or low-intensity loading but becomes stiffer under rapid or high-intensity deformation may provide a biomimetic strategy for adaptive plantar load modulation.

Shear-stiffening gel (STG) materials offer a promising platform for passive stiffness adaptation. Unlike conventional elastomers with relatively constant stiffness, STG exhibits increased apparent stiffness and viscosity under higher deformation rates [40,41,42,43,44]. This rate-dependent response arises from reversible dynamic interactions within the polymer network, particularly boron–oxygen interactions in polyborondimethylsiloxane (PBDMS)-based STG, which permit molecular mobility under slow deformation but restrict chain motion during rapid loading [42,43,44]. Previous studies on shear-stiffening polymers and viscoelastic silicone-based materials have shown that such behavior can be described phenomenologically using Maxwell-type or standard linear solid frameworks, providing a basis for the rate-activated constitutive model adopted in this study [45,46,47]. When embedded within a compliant elastomeric structure, STG may provide conformity during low-demand loading while offering enhanced reinforcement during high-rate events such as heel strike or push-off. Localized placement of STG elements may therefore enable passive, region-specific support within an insole. In this study, the biomimetic concept is limited to material-level imitation of rate-dependent stiffness modulation rather than anatomical replication of the foot.

Inspired by the variable-stiffness behavior of the human foot, this study developed a passive rate-adaptive bioinspired insole (bio-insole) by integrating STG into a Dragon Skin 30 (DS30) silicone elastomer backbone. The STG-containing elastomer composite was first characterized under rate-dependent and cyclic compression. A customized prototype was then evaluated in a preliminary single-case proof-of-concept walking test involving an older adult with sarcopenia, pes cavus, and hindfoot varus tendency. Pressure-derived plantar force, peak pressure, lower-limb electromyography (EMG), and sagittal-plane joint kinematics were compared among the bio-insole, a stock insole, and a rigid insole. This study examined whether an STG-based passive insole can generate localized rate-adaptive mechanical behavior and alter regional plantar loading during walking, while recognizing that the human-subject findings are exploratory and participant-specific.

## 2. Materials and Methods

### 2.1. Bioinspired Design of the Tunable-Stiffness Insole

The rate-adaptive bio-insole was designed as a passive, material-based architecture that translates the loading-rate-dependent stiffness adaptation of the human foot during gait into localized plantar stiffness modulation. Rather than anatomically replicating the foot, the design implemented localized rate-adaptive material elements in plantar regions associated with stance-phase loading.

The bio-insole consisted of a silicone elastomer backbone embedded with STG inserts. The silicone matrix provided baseline structural integrity and shape retention, whereas the STG inserts were intended to provide localized rate-dependent reinforcement under rapid or high-intensity loading. Under low loading rates, the STG-containing regions were expected to remain relatively compliant, allowing plantar conformity and comfort. Under higher loading rates, the apparent stiffness of the STG increases, thereby providing greater local mechanical resistance in regions exposed to rapid loading during gait.

For the present proof-of-concept prototype, the regional layout of the STG inserts was tailored to a single older participant who presented with sarcopenia, pes cavus, and hindfoot varus tendency. These subject-specific features were considered relevant to the design because they may contribute to lateralized rearfoot loading, reduced arch-region plantar contact, impaired active support of the foot–arch complex, and altered load transfer during stance. Accordingly, STG inserts were placed a priori in two functionally relevant regions: the lateral heel and the medial longitudinal arch. This placement was informed by the participant’s foot profile, reported loading characteristics of high-arched/supinated feet, previous arch-support insole concepts [20,21,23], and common orthotic strategies for assisting rearfoot alignment in varus-type foot posture.

**Figure 1:**
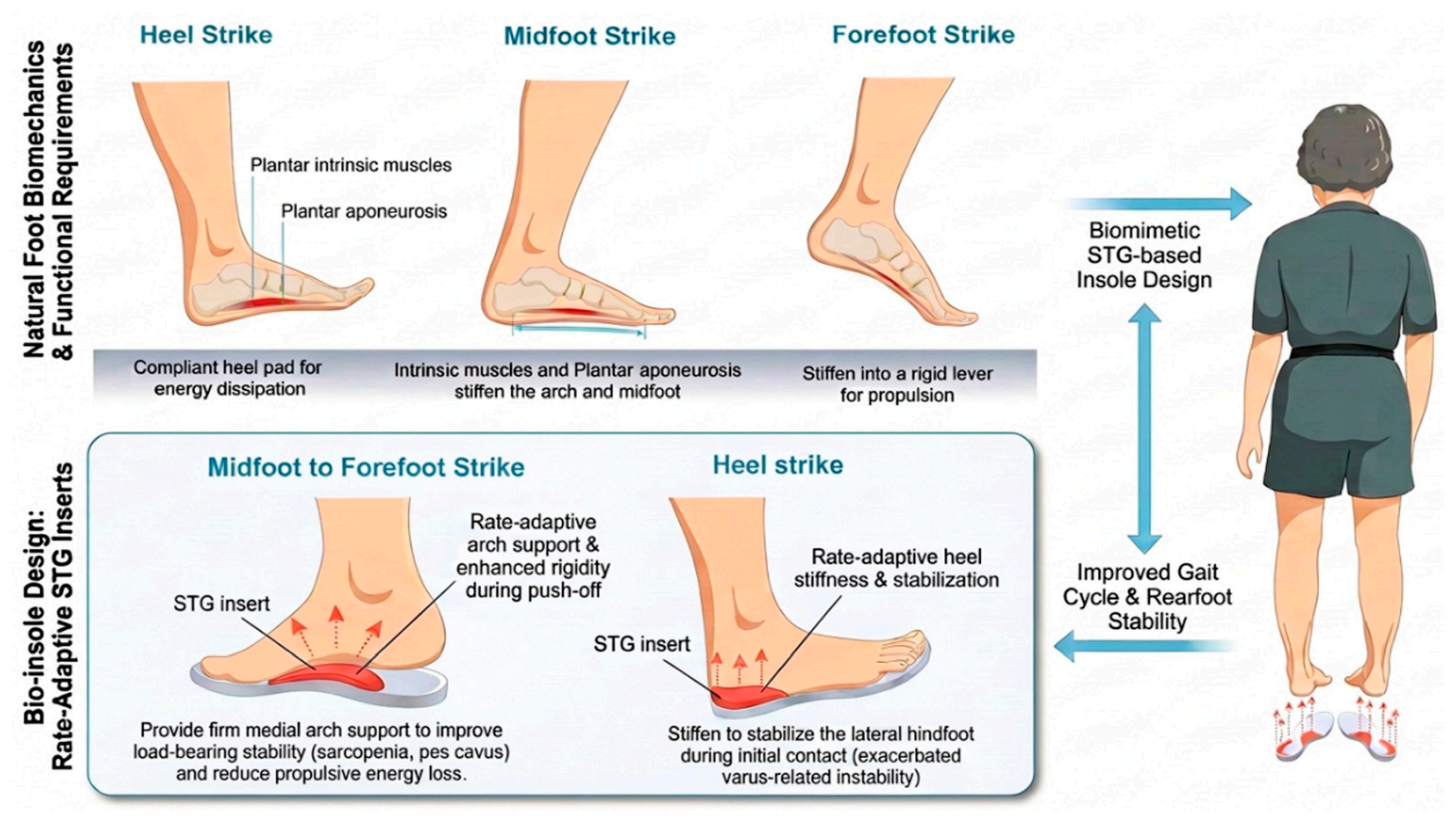
Schematic illustration of bioinspired passive stiffness modulation in the bio-insole, mapped from one functional aspect of gait-dependent stiffness regulation of the human foot.

The lateral heel insert was intended to provide rate-adaptive reinforcement during initial contact, when rapid loading may exacerbate lateralized heel loading and rearfoot instability. The medial arch insert was intended to increase mechanical engagement of the arch region during mid-to-late stance, particularly in the presence of reduced midfoot contact and diminished muscular support. Thus, the regional STG configuration was designed to provide passive, localized stiffness modulation in plantar regions relevant to rearfoot loading, arch support, and stance-phase load transfer. A schematic illustration of the design concept is shown in Figure 1.

### 2.2. Rate-Stiffening Mechanism and Constitutive Description

PBDMS was used as the STG in this study. PBDMS is a lightly cross-linked silicone-based polymer formed by introducing boron-containing cross-links into a polydimethylsiloxane network. Its rate-dependent mechanical behavior is governed by reversible boron–oxygen interactions. Under slow deformation, these transient bonds can dissociate and reform, allowing molecular rearrangement and stress relaxation. Under rapid deformation, bond reconfiguration is restricted, limiting chain mobility and increasing the apparent stiffness and viscous resistance of the material [42,43,44]. This dynamic-bond mechanism provides the material basis for the rate-adaptive stiffening behavior used in the present insole design.

To interpret the rate-stiffening behavior of the STG-containing composite, a rate-activated standard linear solid (RA-SLS) framework was employed as a phenomenological constitutive description informed by the underlying dynamic-bond mechanism. This framework is derived from the classical standard linear solid model, in which an equilibrium spring is connected in parallel with a Maxwell branch [45,46,47]. The equilibrium spring represents the long-term elastic contribution of the elastomeric matrix, whereas the Maxwell branch describes the rate-sensitive viscoelastic response arising from the dynamic polymer network of the STG. In the RA-SLS formulation, the effective modulus and viscosity of the Maxwell branch are allowed to increase with deformation rate, thereby providing a concise mathematical representation of the transition from compliant deformation at low rates to enhanced stiffness under rapid loading. This framework was used to support mechanistic interpretation of the experimental response, rather than to serve as a fully validated predictive constitutive law for all loading conditions.

The engineering strain rate was defined as

(1) $$\dot{\varepsilon }=\frac{v}{h_{0}}$$

where *v* is the compression speed and *h*_0_ is the initial specimen height. In the RA-SLS description, $\dot{\varepsilon }$ serves as the rate variable governing the activation of the rate-sensitive viscoelastic branch. A rate-activation factor was introduced to describe the progressive stiffening of the rate-sensitive branch:

(2) $$\phi (\dot{\varepsilon })=\frac{1}{1+{\left(\frac{{\dot{\varepsilon }}_{c}}{\left|\dot{\varepsilon }\right|}\right)}^{m}}$$

where ${\dot{\varepsilon }}_{c}$ is the characteristic strain rate associated with the onset of rate-dependent stiffening, with units of *s*^−1^, and *m* is a dimensionless sharpness coefficient that controls the steepness of the transition from the low-rate viscoelastic regime to the high-rate stiffened regime. The activation function $\phi (\dot{\varepsilon })$ varies from 0 to 1 for non-zero strain rates, representing the gradual activation of the rate-sensitive branch.

The effective modulus and viscosity of the rate-sensitive Maxwell branch were expressed as

(3) $$E_{1}(\dot{\varepsilon })=E_{1,0}+\Delta E_{1}\phi (\dot{\varepsilon })$$

(4) $$\eta_{1}(\dot{\varepsilon })=\eta_{0}+\Delta \eta \phi (\dot{\varepsilon })$$

where $E_{1}(\dot{\varepsilon })$ is the effective modulus of the rate-sensitive Maxwell branch, *E*_1,0_ is its baseline modulus, and Δ*E*_1_ is the rate-induced modulus increment. Similarly, $\eta_{1}(\dot{\varepsilon })$ is the effective viscosity of the rate-sensitive branch, *η*_0_ is the baseline viscosity, and Δ*η* is the rate-induced viscosity increment. The units of $E_{1}(\dot{\varepsilon })$, *E*_1,0_ and Δ*E*_1_ are Pa, while the units of $\eta_{1}(\dot{\varepsilon })$, *η*_0_ and Δ*η* are Pa·s. Thus, both the elastic and viscous contributions of the Maxwell branch increase progressively as the rate-activation factor increases.

The one-dimensional constitutive relationship of the RA-SLS model can be written as

(5) $$\sigma +\tau (\dot{\varepsilon })\dot{\sigma }=E_{0}\varepsilon +\eta_{1}(\dot{\varepsilon })(1+\frac{E_{0}}{E_{1}(\dot{\varepsilon })})\dot{\varepsilon }$$

where *σ*, *ε*, and $\dot{\varepsilon }$ are the compressive stress, engineering strain, and engineering strain rate, respectively; *E*_0_ is the equilibrium modulus; and the rate-dependent relaxation time is given by

(6) $$\tau (\dot{\varepsilon })=\frac{\eta_{1}(\dot{\varepsilon })}{E_{1}(\dot{\varepsilon })}$$

For small-amplitude harmonic loading at angular frequency *ω*, evaluated at a given characteristic strain-rate state, the corresponding complex modulus is

(7) $$E^{*}(\omega ,\dot{\varepsilon })=E_{0}+\frac{E_{1}(\dot{\varepsilon })\mathrm{i\omega \tau}(\dot{\varepsilon })}{1+\mathrm{i\omega \tau}(\dot{\varepsilon })}$$

The storage modulus *E*′ and loss modulus *E*″ are therefore expressed as

(8) $$E^{\prime }(\omega ,\dot{\varepsilon })=E_{0}+\frac{E_{1}(\dot{\varepsilon }){\left[\omega \tau (\dot{\varepsilon })\right]}^{2}}{1+{\left[\omega \tau (\dot{\varepsilon })\right]}^{2}}$$

(9) $$E^{\prime \prime }(\omega ,\dot{\varepsilon })=\frac{E_{1}(\dot{\varepsilon })\omega \tau (\dot{\varepsilon })}{1+\left[\omega \tau (\dot{\varepsilon })\right]{)}^{2}}$$

Here, *E*′ denotes the storage modulus associated with elastic energy storage, and *E*″ denotes the loss modulus associated with viscous energy dissipation. A higher *E*′ indicates greater dynamic stiffness, whereas a higher *E*″ indicates greater damping capacity. In this framework, increasing strain rate enhances $\phi (\dot{\varepsilon })$, which increases both $E_{1}(\dot{\varepsilon })$ and $\eta_{1}(\dot{\varepsilon })$. This coupled increase provides a phenomenological explanation for the rate-adaptive stiffening and energy-dissipating behavior of the STG-containing composite under plantar-loading-relevant conditions.

### 2.3. Materials and Specimen Fabrication

DS30 silicone elastomer (Smooth-On, Macungie, PA, USA) was selected as the primary structural material for the bio-insole because of its relatively high baseline stiffness, elasticity, and geometric stability. DS30 served as the load-bearing matrix, whereas STG provided rate-dependent stiffening in selected regions. Ecoflex 00-50 silicone elastomer (EC50; Smooth-On, Macungie, PA, USA), which has a lower baseline modulus, was used to fabricate comparison specimens in order to evaluate the influence of matrix stiffness on the rate-dependent response of the STG composite.

The PBDMS-based STG was synthesized by introducing boric acid into hydroxyl-terminated PDMS, as described previously with minor adaptation. Briefly, boric acid was mixed with hydroxyl silicone oil at 30 mg/mL, stirred until homogeneous, gradually heated to 180 °C, and maintained under continuous stirring for 1 h. The mixture became viscous and sticky during heating due to boron–silicone dynamic crosslink formation. After cooling to room temperature, the obtained PBDMS-based STG was used for subsequent composite fabrication [43].

Representative sandwich specimens were fabricated to evaluate the compressive behavior of the insole material system. Each specimen had a cylindrical geometry with a diameter of 37 mm and a total height of 16.5 mm. This geometry provided a reproducible material-level configuration for controlled uniaxial compression testing. The sandwich specimens were used to characterize the basic compressive response of the DS30–STG composite system and were not intended to replicate the exact geometry or boundary conditions of the final foot-shaped insole.

**Figure 2:**
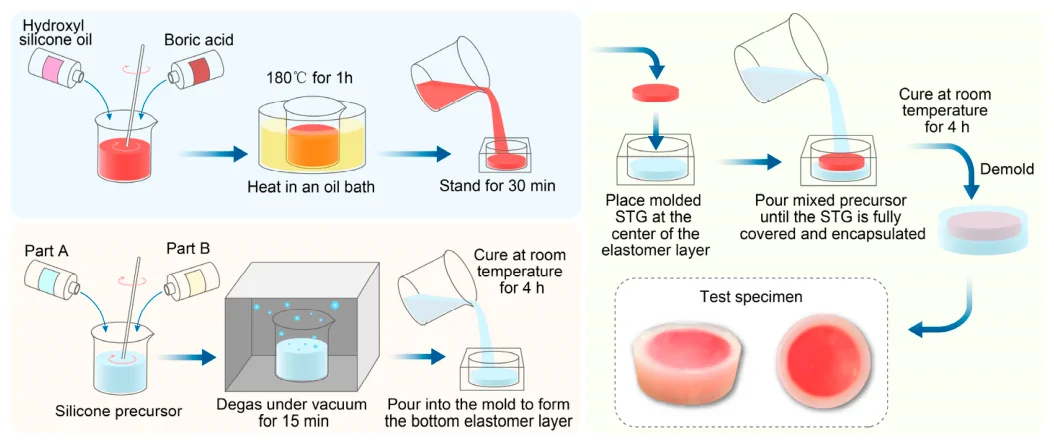
Fabrication process of the DS30-STG sandwich specimen used for material-level compression testing.

For DS30-STG specimens, DS30 precursor components A and B were mixed at a 1:1 mass ratio, thoroughly stirred, and degassed under vacuum for 15 min to remove entrapped air bubbles. The mixture was first poured into a 3D-printed cylindrical mold to form the bottom elastomer layer and cured at room temperature for 4 h. Separately, STG was pre-shaped in a cylindrical mold with a diameter of 30 mm and a height of 10 mm. The pre-shaped STG insert was then placed onto the cured DS30 bottom layer. Fresh DS30 precursor was subsequently poured into the mold to fully encapsulate the STG. After complete curing, a DS30-STG-DS30 sandwich specimen, referred to as the DS30-STG specimen, was obtained (Figure 2).

EC50-STG specimens were fabricated using the same geometry and procedure, except that EC50 was used as the encapsulating elastomer instead of DS30. Monolithic DS30 specimens with the same outer dimensions were also prepared as controls. The fabricated specimen types were therefore DS30, DS30-STG, and EC50-STG.

### 2.4. Mechanical Characterization

Uniaxial compression tests were performed to evaluate the rate-dependent mechanical response of the fabricated specimens. All tests were conducted at room temperature using a universal testing machine equipped with a 500 N load cell (EZ-LX HS, Shimadzu, Kyoto, Japan). Each specimen was placed centrally between two parallel circular compression plates to ensure coaxial loading. Compression tests were performed at four crosshead speeds: 30, 300, 1000, and 2000 mm/min, corresponding to nominal engineering strain rates of approximately 0.03, 0.30, 1.01, and 2.02 s^−1^, respectively, based on the initial specimen height of 16.5 mm. These loading rates were selected to examine the rate-stiffening behavior of the STG-containing composites within the operating range of the testing system. One specimen was tested three times under each condition; therefore, the repeats represent technical rather than independent specimen replicates.

The compressive stress was calculated by dividing the measured force by the initial cross-sectional area of the specimen. The engineering strain was calculated as the displacement divided by the initial specimen height. The apparent compressive modulus was determined from the average slope of the stress–strain curve within a selected strain range. For DS30 and DS30-STG specimens, the modulus was calculated within the 5–25% strain range. For EC50-STG specimens, the modulus was calculated within the 20–40% strain range because of their lower baseline stiffness and larger deformation under compression.

Cyclic compression tests were further conducted to assess the repeatability and short-term mechanical stability of the DS30-STG structure. Specimens were compressed to a nominal strain of 40% for 100 loading–unloading cycles at compression speeds of 30 and 1000 mm/min. These two rates were selected to represent low-rate and high-rate loading conditions, respectively. The cyclic stress–strain curves were recorded to evaluate mechanical repeatability, hysteresis, and possible degradation of the sandwich structure during repeated deformation.

### 2.5. Fabrication of the Customized Bio-Insole

A customized bio-insole was fabricated according to the participant-specific design rationale described in Section 2.1. The insole consisted of a DS30 elastomeric backbone with regionally embedded STG inserts. The overall thickness of the bio-insole was approximately 5 mm, which was designed with reference to the stock insole to maintain shoe compatibility and underfoot comfort. The STG inserts used in the wearable bio-insole were different from the 10 mm thick specimens used for material-level sandwich compression testing in Section 2.3. In the wearable prototype, thinner STG inserts were shaped according to the target plantar regions and embedded within the approximately 5 mm insole structure.

The bio-insole was fabricated using the same encapsulation-based molding process used for the sandwich specimens. A bottom DS30 layer was first cast in an insole-shaped mold and partially cured. The pre-shaped STG inserts were then placed at the predefined plantar locations, after which fresh DS30 precursor was poured to fully encapsulate the inserts and form the final insole geometry. The insole was cured at room temperature until complete solidification and then trimmed to fit the participant’s shoe.

### 2.6. Wearable Evaluation Protocol

A preliminary single-case proof-of-concept walking experiment was conducted to evaluate the wearable performance of the customized STG-based bio-insole under subject-specific walking conditions. The participant was a 77-year-old woman with a body mass of 39.05 kg and a height of 1.42 m. She was selected from a previous study cohort because she presented with a biomechanical profile relevant to the intended design application, including sarcopenia, pes cavus and hindfoot varus tendency.

The participant had previously been classified as having sarcopenia within the source cohort according to the Asian Working Group for Sarcopenia 2019 (AWGS 2019) algorithm [48]; the individual diagnostic measurements were not reassessed during the present experiment. Static foot posture was assessed using the Foot Posture Index-6 (FPI-6), with an FPI-6 score of −4 indicating a mild degree of supination [49,50]. Quiet-standing plantar morphology further showed reduced midfoot contact, consistent with a high-arched/pes cavus foot morphology. Hindfoot varus tendency was assessed clinically based on posterior standing observation, genu varum alignment, mildly supinated foot posture, and lateral heel shoe wear. Because no radiographic or quantitative hindfoot alignment measurement was performed, hindfoot varus is described in this study as a clinically observed tendency rather than a quantified structural diagnosis.

This participant was considered suitable for an initial subject-specific feasibility assessment because the coexistence of reduced muscular capacity, elevated medial longitudinal arch, reduced midfoot contact, and varus-type rearfoot alignment may increase the need for plantar load redistribution, arch-region mechanical engagement, and rearfoot stabilization during walking. The wearable evaluation was therefore intended to examine the feasibility of applying a subject-specific STG-based bio-insole in an individual with relevant foot biomechanical deficits, rather than to establish population-level efficacy.

The participant had previously participated in the Intelligent Robotics for Elderly Assistance in Hong Kong project, supported by the Research Grants Council of the Hong Kong Special Administrative Region, China (Grant No. T42-717/20-R; ethics approval No. EA1903040). The present study was approved by the Human Research Ethics Committee of The University of Hong Kong (HREC approval No. EA220339). Written informed consent was obtained before testing, and all procedures were conducted in accordance with the approved protocol.

**Figure 3:**
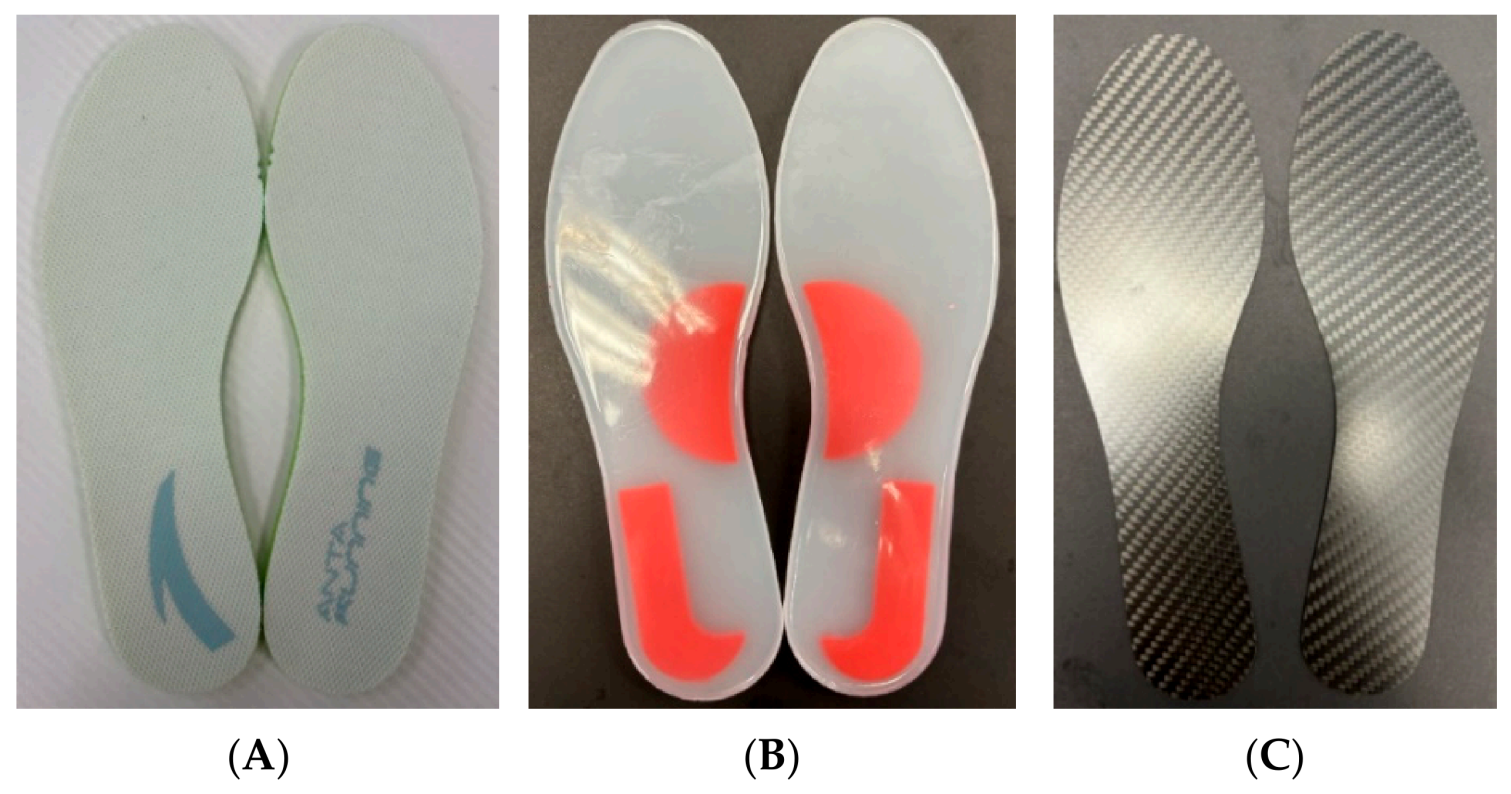
Three different types of insoles evaluated in this investigation: (**A**) stock insole, (**B**) bio-insole, and (**C**) rigid insole. All insoles were shoe size 37, corresponding to an approximate foot length of 235 mm. The stock insole and bio-insole had similar thicknesses of approximately 5 mm, whereas the commercially available carbon-fiber rigid insole had a thickness of approximately 1.2 mm.

Three insole conditions were evaluated: the stock insole originally provided with the shoe, the customized STG-based bio-insole, and a commercially available rigid carbon-fiber insole, as shown in Figure 3. For the bio-insole and rigid-insole conditions, the original stock insole was removed before testing. The stock insole served as the baseline control. The bio-insole was fabricated with reference to the stock insole in both size and thickness, and both had an approximate thickness of 5 mm. All three insoles had the same nominal shoe size (size 37), corresponding to an approximate foot length of 235 mm. The rigid carbon-fiber insole had the same nominal size but was thinner, with an approximate thickness of 1.2 mm. Although the three insoles had similar overall outline dimensions, the actual plantar contact area and shoe–insole interaction were not quantified and may have differed across conditions. The rigid insole was included as a practical fixed-stiffness comparator because carbon-fiber-based rigid inserts have been reported to alter footwear bending stiffness, forefoot lever function, and plantar pressure distribution. Thus, this comparison was intended to provide an initial practical reference between the proposed localized rate-dependent bio-insole and a commonly used fixed-stiffness insole condition. However, the rigid insole also differed from the stock and bio-insole conditions in thickness, material composition, surface conformity, and likely effective in-shoe interaction. Therefore, stiffness was not isolated as a single experimental factor, and differences between the rigid insole and bio-insole conditions should be interpreted as reflecting multiple design variables rather than rate-dependent versus fixed stiffness alone.

All walking tests were conducted using the same pair of lightweight commercial running shoes with removable insoles. For this single-participant proof-of-concept evaluation, the order of the three insole conditions was randomized to reduce systematic sequencing bias. However, because only one participant was tested, this procedure could not statistically control for potential order effects, and fatigue, familiarization, or carryover effects between conditions cannot be excluded. Blinding of the participant and experimenters was not feasible because the stock, bio-insole, and rigid insole differed in material properties, stiffness, appearance, and perceived underfoot feel.

Before data collection under each condition, the participant completed a 20 min familiarization walk with the corresponding insole. She then performed three trials of a 4 m straight walking task at a self-selected comfortable speed, with 1 min of rest between trials. Self-selected speed was used to prioritize participant safety and preserve a natural walking pattern under each insole condition. The mean walking speeds were 0.56, 0.60, and 0.63 m/s for the stock-insole, bio-insole, and rigid-insole conditions, respectively. Because speed was not experimentally controlled, these small between-condition differences were treated as a potential confounding factor in the interpretation of plantar loading, EMG, and kinematic outcomes. Although they may also reflect factors such as comfort, familiarization, or perceived stability/confidence, no direct measures of balance, postural stability, perceived stability, confidence, or fall risk were collected; therefore, walking speed was not interpreted as a surrogate for these outcomes.

Safety and comfort were monitored throughout the wearable evaluation because the participant was an older adult with low body weight and foot deformity. Fall-prevention measures were implemented during both preparation and walking trials. During sensor placement, bilateral handrails were positioned beside the participant to reduce the risk of instability or falling. Insoles were changed while the participant remained seated. During the 4 m walking trials, handrail sections were placed along both sides of the walkway to provide immediate support if needed. In addition to the experimental operators, two helpers were present throughout the session to monitor walking stability and provide assistance if any sign of imbalance or fall risk occurred.

Foot condition and comfort were also monitored during insole fitting and walking. The participant’s feet were visually inspected, and she was asked to report any pain, discomfort, or abnormal underfoot sensation. No obvious skin abnormality was observed, and the participant did not report clear foot pain or discomfort during the testing session. After completing the experiment, the participant completed an Acceptance Questionnaire for Biomimetic Insoles using a 0–6 scale, where 0 indicated “not at all” and 6 indicated “a great deal.” The participant rated the overall comfort of the bio-insole as 5/6, and region-specific comfort scores were also 5/6 for the forefoot, midfoot, heel, and hallux regions.

### 2.7. Data Acquisition

Plantar force and pressure data were collected at 100 Hz using a medilogic WLAN instrumented insole system (Medilogic, Schönefeld, Germany), integrated with the Noraxon data acquisition platform. Pressure-derived vertical plantar force and plantar pressure distributions were exported from the raw data files for subsequent analysis.

Lower-limb kinematics were recorded simultaneously using a 16-sensor Xsens inertial motion capture system at 60 Hz. Sensors were attached to the head, shoulders, sternum, wrists, hands, pelvis, thighs, shanks, and dorsum of both feet according to the manufacturer’s recommended protocol. The Xsens MVN data were processed using the Movella software toolkit (version 2024.2.1) and exported in CSV format to obtain sagittal-plane ankle and knee joint angles. The complete wearable sensor setup, including the Xsens sensors, surface EMG electrodes, EMG recording device, and medilogic WLAN insoles, is shown in Supplementary Figure S3.

A supplementary foot-velocity assessment was conducted separately in a healthy adult volunteer as a preliminary design-stage contextual check before fabrication of the bio-insole. Given the rate-dependent stiffening observed in material testing, this assessment was intended only to determine whether locomotion includes transient foot-segment motion of a magnitude relevant to the feasibility of a rate-adaptive STG-based insole concept. It was not part of the main walking trials performed by the older participant. Using the same Xsens system and processing workflow, the horizontal and vertical foot-segment velocity components were extracted as Vx and Vz, respectively, during slow walking, normal walking, fast walking, and jogging (Supplementary Figure S4). These measurements provided only contextual information and were not interpreted as direct estimates of local compression, shear, or deformation rate within the embedded STG inserts.

Surface EMG signals were acquired at 1000 Hz using a custom-built recording device integrated with a multichannel acquisition card (SMACQ USB3000 series, SMACQ, Beijing, China) [51,52]. Six muscles on each lower limb were monitored: tibialis anterior (TA), lateral gastrocnemius (GL), vastus lateralis, rectus femoris, semitendinosus, and biceps femoris. In the present analysis, the right TA and right GL were selected because of their primary roles in ankle control and propulsion during walking, and the right side was analyzed for consistency with the right-foot plantar pressure analysis and the participant-specific insole design.

Raw EMG signals were processed in MATLAB (R2020b). The direct current (DC) offset was removed by subtracting the mean value of each raw signal. The demeaned signals were band-pass filtered between 20 and 450 Hz using a third-order Butterworth filter with zero-phase forward–reverse filtering. The filtered signals were then full-wave rectified, and linear envelopes were obtained by low-pass filtering at 4 Hz using a third-order Butterworth filter with zero-phase forward–reverse filtering. For temporal normalization, each gait cycle was resampled to 0–100% of the stance phase. In the present study, the EMG analysis focused on the stance phase because the insole mechanically interacts with the foot primarily during foot–ground contact, particularly during initial contact, midstance, and push-off, whereas its direct contribution after toe-off is minimal. In addition, muscle activity during swing is less directly related to plantar support and load redistribution. Therefore, only stance-phase EMG profiles were extracted, normalized, and averaged across trials for further comparison.

Amplitude normalization based on maximum voluntary contraction (MVC) was not performed. Therefore, EMG analysis was limited to within-participant, within-muscle comparisons across insole conditions. Electrode placement, acquisition settings, and processing procedures were maintained consistently across all walking conditions. The processed EMG linear-envelope amplitudes were directly compared to evaluate relative changes in TA and GL activity under different insole conditions. Because EMG amplitudes were not normalized to MVC or another reference contraction, the results should not be interpreted as absolute muscle activation levels or compared across different muscles.

### 2.8. Data Processing and Outcome Measures

**Figure 4:**
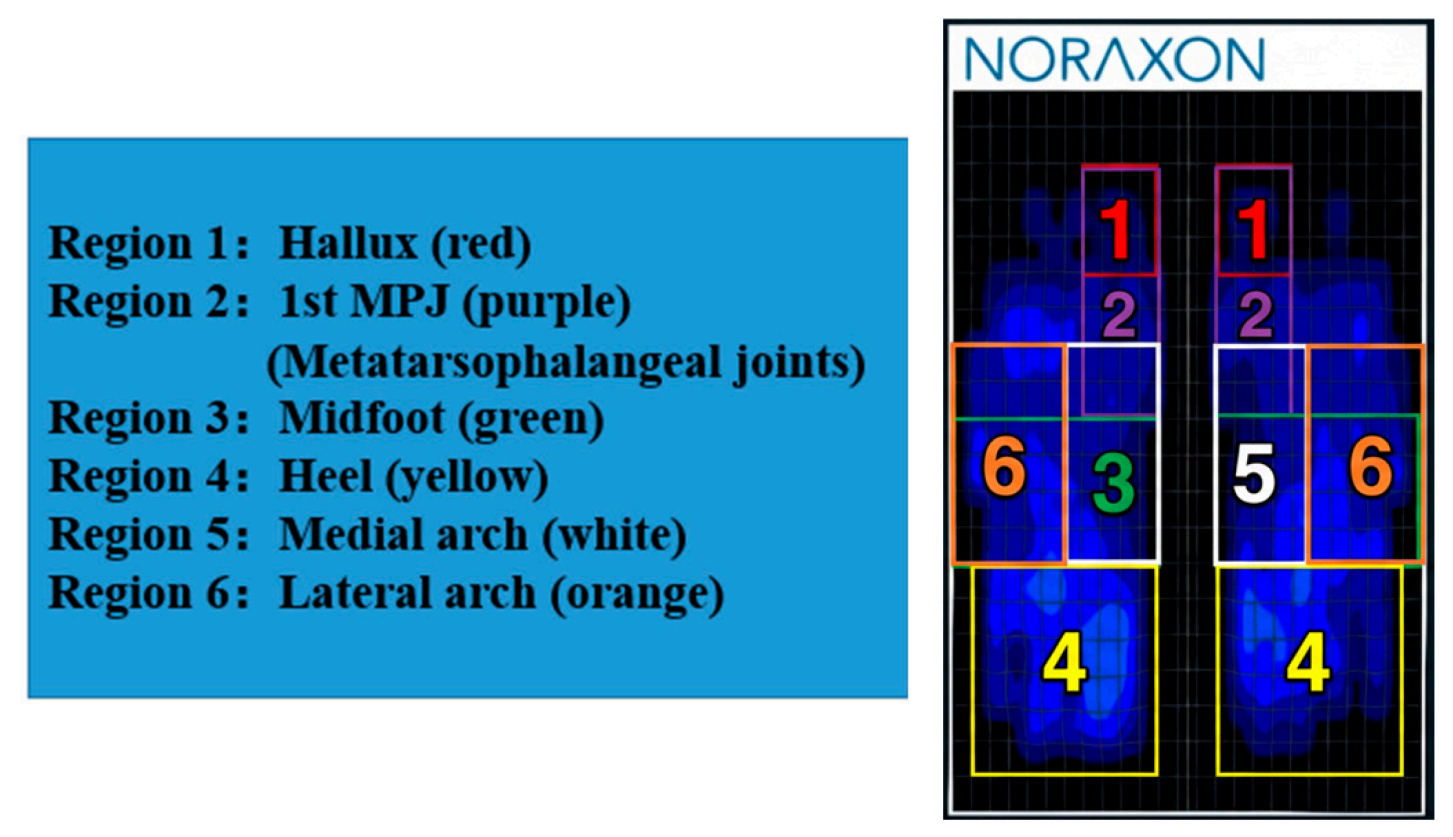
Offline segmentation of the exported medilogic WLAN insole 20 × 12 coordinate-grid plantar pressure matrix into six anatomical regions for analysis of regional force and peak pressure. The six regions were heel, midfoot, medial arch, lateral arch, 1st MPJ, and hallux.

The plantar pressure distribution was exported from Noraxon MR3 software (version 3.18) as the medilogic WLAN insole system’s 20 × 12 coordinate-grid pressure matrix and segmented offline into six anatomical regions: heel, midfoot, medial arch, lateral arch, first metatarsophalangeal joint (1st MPJ), and hallux (Figure 4). Because this 20 × 12 grid provides limited spatial resolution for small anatomical regions, the hallux—and, to a lesser extent, the 1st MPJ—may be represented by relatively few sensels. Therefore, very low regional values should be interpreted cautiously, as they may partly reflect spatial-resolution and low-load sensitivity limits rather than true absence of loading. The segmentation was based on anatomically and functionally relevant plantar-loading areas, with reference to previously reported plantar-region segmentation approaches [18,19]. Only right-foot data were analyzed for consistency with the participant-specific insole design and regional loading assessment.

The six regions represented key functional loading areas during walking. The heel region was used to characterize early-stance heel loading, the midfoot and arch regions were used to assess midstance plantar support, and the 1st MPJ and hallux regions were used to evaluate late-stance forefoot loading and push-off. Because the bio-insole incorporated STG inserts in the lateral heel and medial arch regions, heel- and arch-related pressure changes were of particular interest.

Stance periods were identified from the right-foot pressure-derived vertical plantar force curve, from force onset after foot contact to force disappearance at toe-off. Because no external force plate was used, gait events and stance phases were operationally defined using the insole-derived force curve and corresponding plantar pressure maps. Each stance phase was time-normalized to 0–100%.

For regional outcome extraction, stance was divided into three predefined windows: early stance, 0–30%; midstance, 30–60%; and late stance, 60–100% of stance. Heel-region outcomes were extracted within early stance, midfoot and arch-region outcomes within midstance, and 1st MPJ and hallux outcomes within late stance. Within each relevant window, regional plantar force and peak pressure were identified using the same procedure across all insole conditions. The corresponding plantar pressure maps were inspected to confirm that the extracted values represented the expected anatomical loading regions.

For each condition, 15 valid right-foot steps, comprising five steps from each of three walking trials, were analyzed for plantar force and pressure after excluding steps with obvious sensor artifacts, incomplete foot contact, irregular acceleration/deceleration patterns, or clear start- or stop-related effects at the beginning or end of the 4 m walkway.

For EMG analysis, stance-phase data were time-normalized and averaged across valid steps. Peak linear-envelope amplitudes of the right TA and right GL were extracted for comparison among insole conditions. For kinematic analysis, sagittal-plane ankle and knee angle trajectories during stance were time-normalized, and stance-phase range of motion (ROM) was calculated for each condition.

Because the wearable evaluation was conducted as a preliminary single-participant proof-of-concept test, data were analyzed descriptively. Results are reported as mean ± standard deviation (SD). Repeated steps were used only to summarize within-participant variability and were not treated as independent biological replicates. No inferential statistical analysis was performed. Therefore, the wearable findings should be interpreted as preliminary, participant-specific observations rather than population-level evidence. Percentage labels and angular-excursion labels in the wearable result figures are provided as descriptive visual aids and should not be interpreted as indicators of statistical significance.

## 3. Results and Discussion

### 3.1. Rate-Dependent Compressive Response of the STG-Based Composite

**Figure 5:**
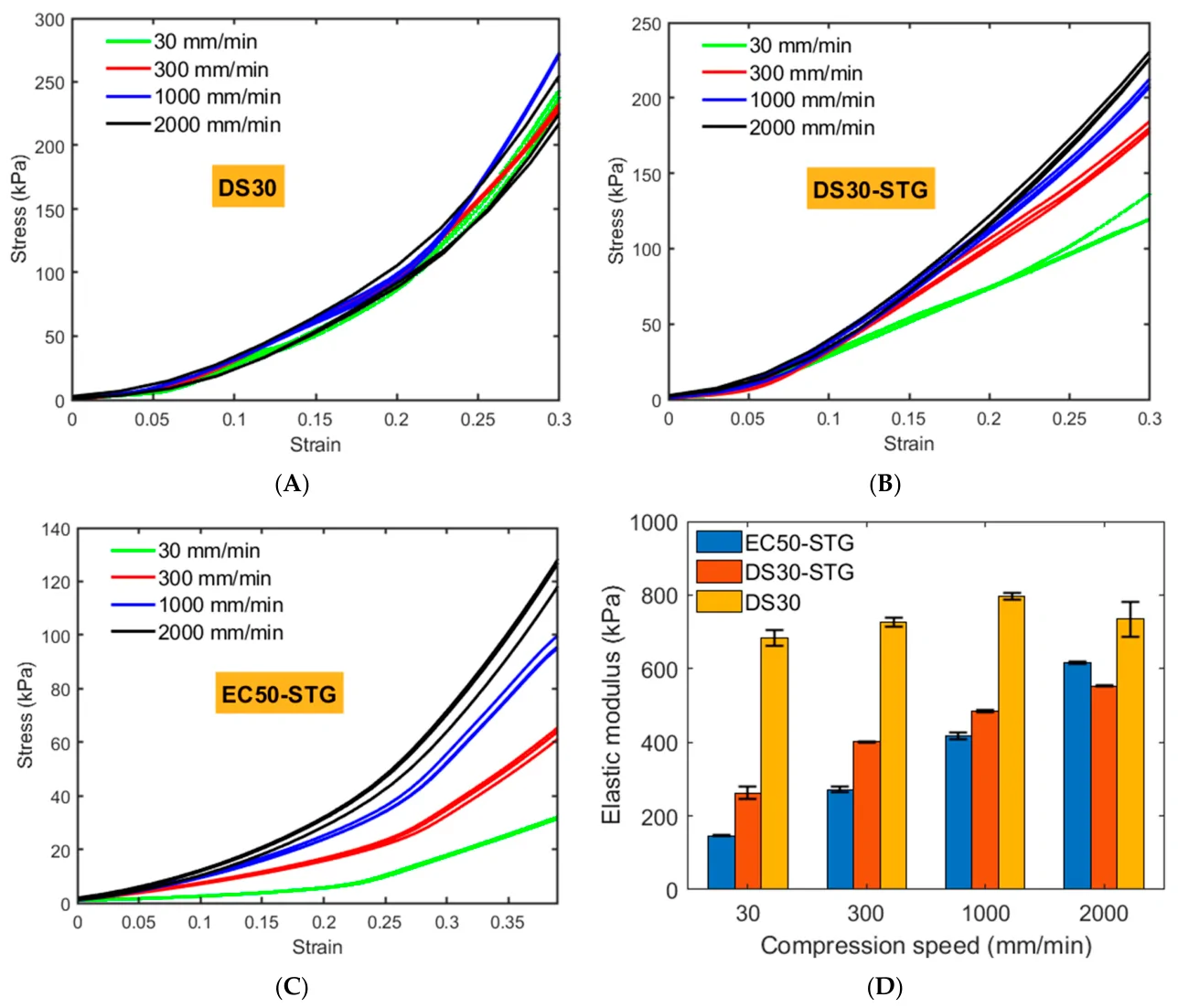
Mechanical properties of DS30, DS30-STG, and EC50-STG specimens at different compression rates. Compressive stress–strain curves for (**A**) DS30, (**B**) DS30-STG, and (**C**) EC50-STG. (**D**) Comparison of the apparent compressive modulus of DS30, DS30-STG, and EC50-STG across compression rates. Error bars represent SD based on three replicates. The corresponding numerical modulus values are provided in Supplementary Table S1.

The compressive behavior of the elastomeric matrix and STG-containing composites was first characterized to identify the material configuration most suitable for the proposed rate-adaptive plantar-support concept. As shown in Figure 5A, DS30 displayed a typical nonlinear elastomeric response with limited loading-rate sensitivity. Its apparent compressive modulus remained approximately constant across the tested compression speeds (about 735 kPa), indicating stable baseline stiffness but minimal intrinsic rate adaptivity.

In contrast, incorporating STG into the DS30 matrix produced a clear rate-dependent stiffening response (Figure 5B). The apparent compressive modulus of the DS30-STG composite, calculated from the linear fitting within the 5–25% strain range, increased from 262.11 kPa at 30 mm/min to 401.74, 485.66, and 553.00 kPa at 300, 1000, and 2000 mm/min, respectively. This progressive increase indicates that the embedded STG phase contributed a rate-sensitive mechanical response to the composite. This behavior is consistent with the design objective of the bio-insole: remaining relatively compliant under slower loading while becoming stiffer under more rapid deformation. It should be noted that embedding the compliant STG core reduced the apparent composite modulus relative to monolithic DS30 at all tested rates. Therefore, “stiffening” in the present study refers to the increase in the DS30-STG modulus with increasing deformation rate, rather than reinforcement beyond the stiffness of monolithic DS30.

A more pronounced rate-dependent response was observed for EC50-STG (Figure 5C). Its apparent compressive modulus, evaluated within the 20–40% strain range, increased from 146.03 kPa at 30 mm/min to 271.09, 416.56, and 616.78 kPa at 300, 1000, and 2000 mm/min, respectively. The comparison among DS30, DS30-STG, and EC50-STG is summarized in Figure 5D. The detailed apparent compressive modulus values for DS30, DS30-STG, and EC50-STG at each compression speed are provided in Supplementary Table S1 and are reported as mean ± SD based on three replicates. The larger relative stiffening observed in EC50-STG may reflect the lower baseline stiffness of the EC50 matrix, which likely permits greater deformation of the encapsulated STG. By contrast, the stiffer DS30 matrix may partially constrain STG deformation and thereby moderate the rate-dependent contribution.

Although EC50-STG exhibited a stronger relative stiffening effect, the low baseline modulus of EC50 may limit its suitability as a load-bearing backbone for an insole intended to support stable plantar support, particularly for an older user with altered foot biomechanics. For wearable plantar support, the material system must provide both sufficient structural integrity and adaptive reinforcement. DS30-STG therefore provided a more appropriate balance: DS30 maintained geometric stability and baseline support, whereas STG introduced rate sensitivity into the composite, although the apparent modulus of DS30-STG remained below that of monolithic DS30 over the tested rate range.

To provide locomotion-related context for the material-level rate-dependent response, foot-segment velocities were analyzed during slow walking, normal walking, fast walking, and jogging. Supplementary Figure S4 shows repeated transient velocity fluctuations across all gait conditions. The vertical velocity component (Vz) displayed distinct peaks around loading-related events, increasing from ~0.8 m/s in slow walking to ~1.1 m/s in normal walking and ~1.2–1.3 m/s in fast walking and jogging. These observations support the presence of rapid foot-motion events during gait that are relevant to considering a rate-sensitive insole concept. Nevertheless, these segment-level measurements should not be interpreted as direct estimates of the local deformation rate within the embedded STG inserts.

### 3.2. Cyclic Mechanical Stability of the DS30-STG Structure

**Figure 6:**
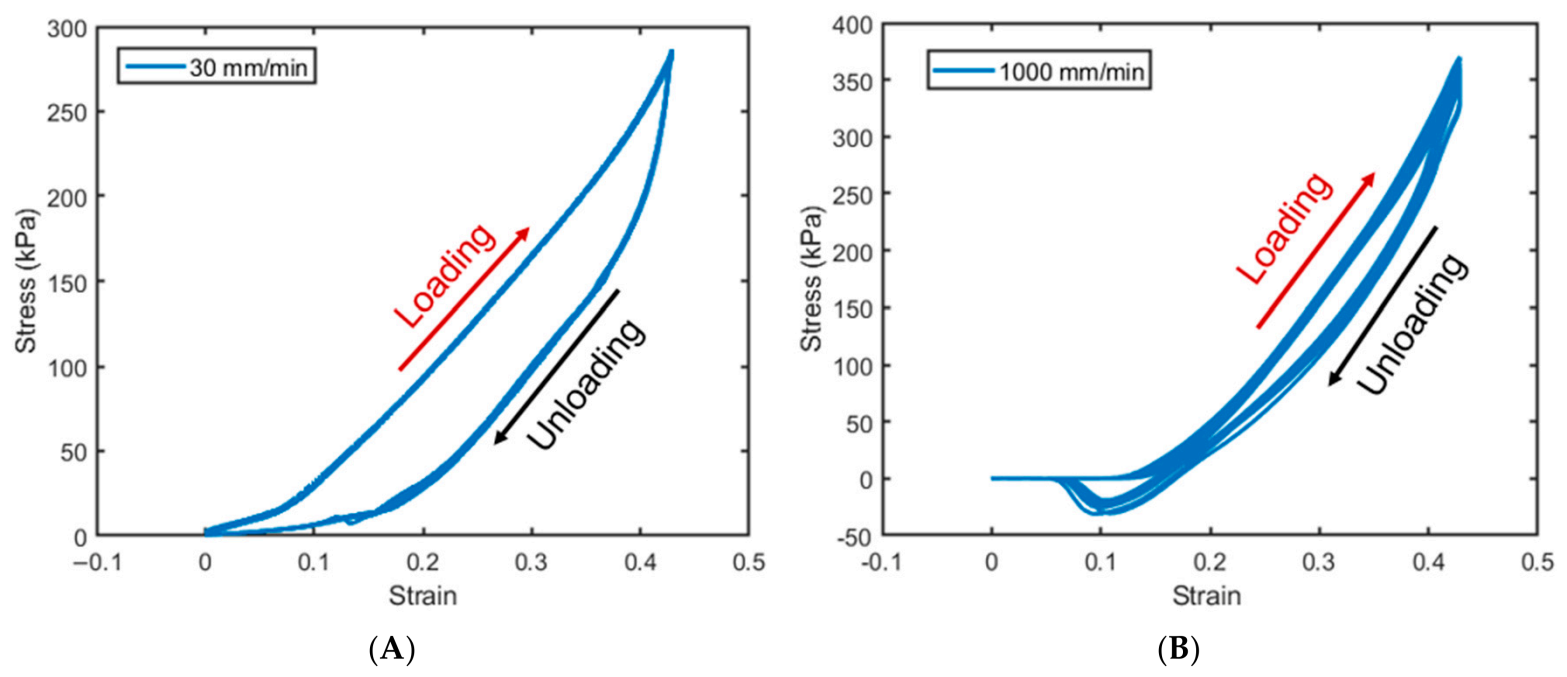
Cyclic compression responses of DS30-STG specimens at 40% strain under compression rates of (**A**) 30 mm/min and (**B**) 1000 mm/min. The curves represent 100 consecutive loading–unloading cycles. The red and black arrows indicate the loading and unloading directions, respectively.

Cyclic mechanical stability is a critical requirement for insole materials because plantar-support elements are subjected to repeated compression during walking. The cyclic compression responses of the DS30-STG sandwich specimens are shown in Figure 6A,B. Under repeated loading to 40% strain, the loading–unloading curves remained qualitatively similar over 100 cycles at both 30 and 1000 mm/min, indicating qualitative short-term repeatability and no obvious mechanical deterioration within the tested range.

At 30 mm/min, the cyclic stress–strain curves showed close overlap across successive cycles, showing no visually apparent gross change in hysteresis within the tested cycle range and limited cycle-dependent softening. At 1000 mm/min, the specimens also retained repeatable mechanical responses, indicating that the composite structure remained intact under high-rate cyclic compression. The overall hysteresis pattern remained stable across cycles, suggesting consistent energy-dissipation behavior within the tested cycle range.

These results indicate that DS30-STG can withstand repeated large-strain compression within the tested cycle range while maintaining stable mechanical behavior and no obvious structural deterioration. When considered together with the monotonic compression results, the cyclic data suggest that DS30-STG is capable of providing both adaptive stiffness modulation and mechanically repeatable performance under repetitive loading conditions relevant to gait. However, because the present cyclic evaluation was limited to 100 cycles, further long-term fatigue testing will be needed to determine its durability under extended daily use.

### 3.3. Preliminary Single-Case Wearable Evaluation of the Bio-Insole

**Figure 7:**
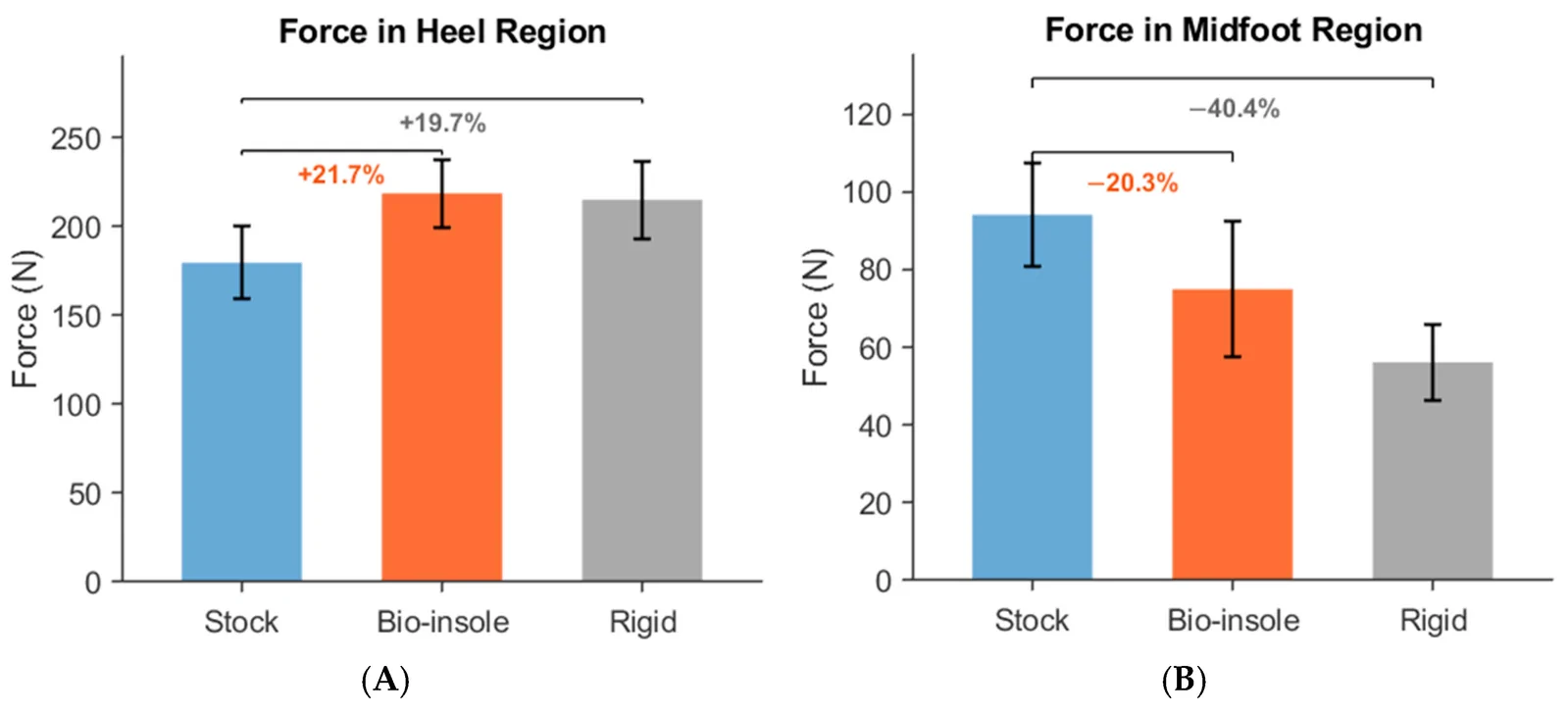

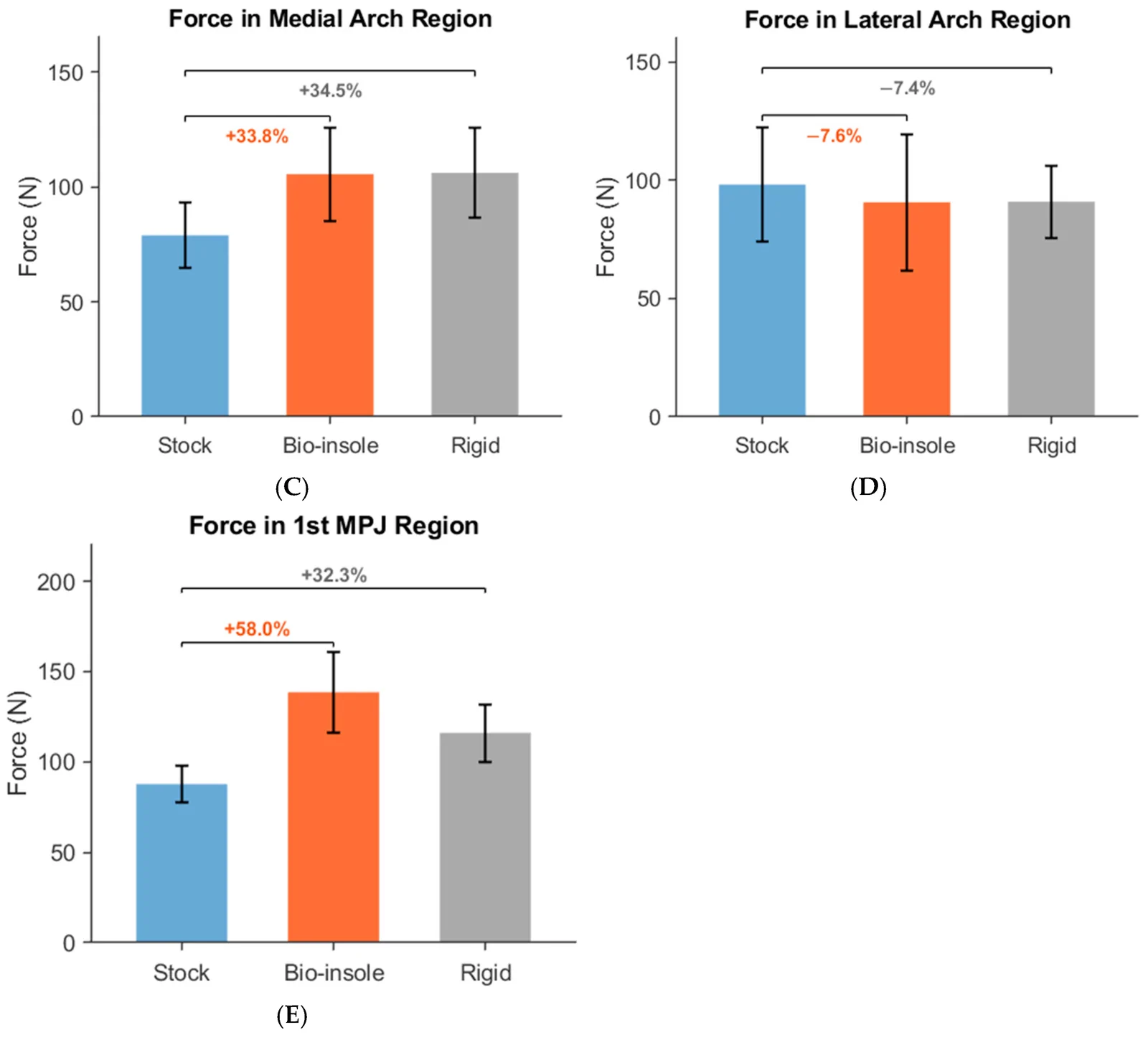
Regional plantar forces during walking under the three insole conditions. Data are presented as mean ± SD. (**A**) Heel. (**B**) Midfoot. (**C**) Medial arch. (**D**) Lateral arch. (**E**) 1st MPJ. Percentage labels indicate descriptive changes relative to the stock-insole condition. These labels are provided for visual comparison among insole conditions and do not represent statistical significance. Hallux values are listed in Table 1 but are not plotted because loading was near zero in most conditions.

**Figure 8:**
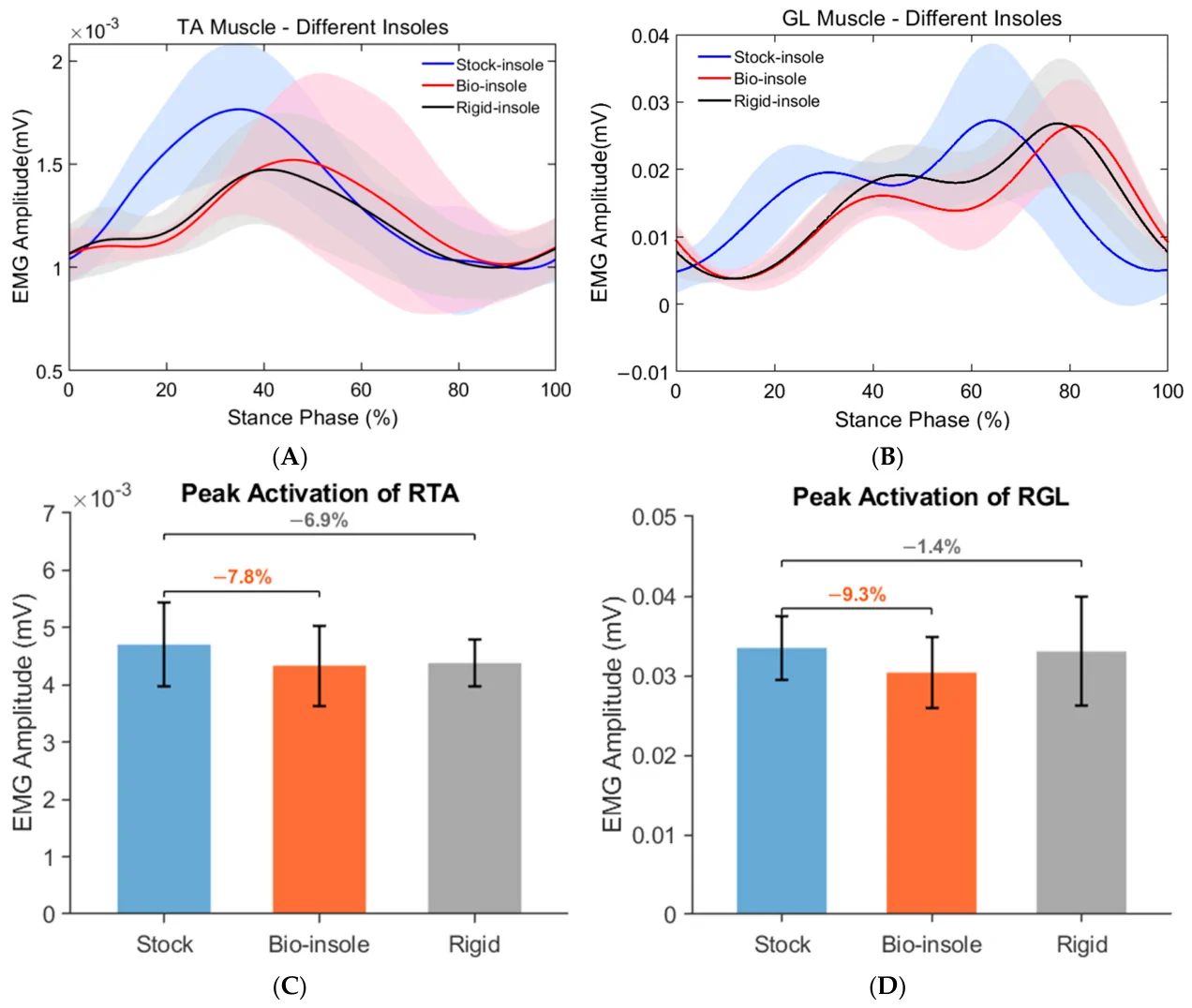
Lower-leg muscle activation during 4 m walking under different insole conditions. Time-normalized EMG profiles during the stance phase are shown for the (**A**) right TA and (**B**) right GL. Solid lines represent mean EMG amplitude, and shaded regions indicate SD. Peak EMG activation of the (**C**) TA and (**D**) GL is presented as mean ± SD. Percentage labels in panels (**C**,**D**) indicate descriptive changes relative to the stock-insole condition and do not represent statistical significance.

**Figure 9:**
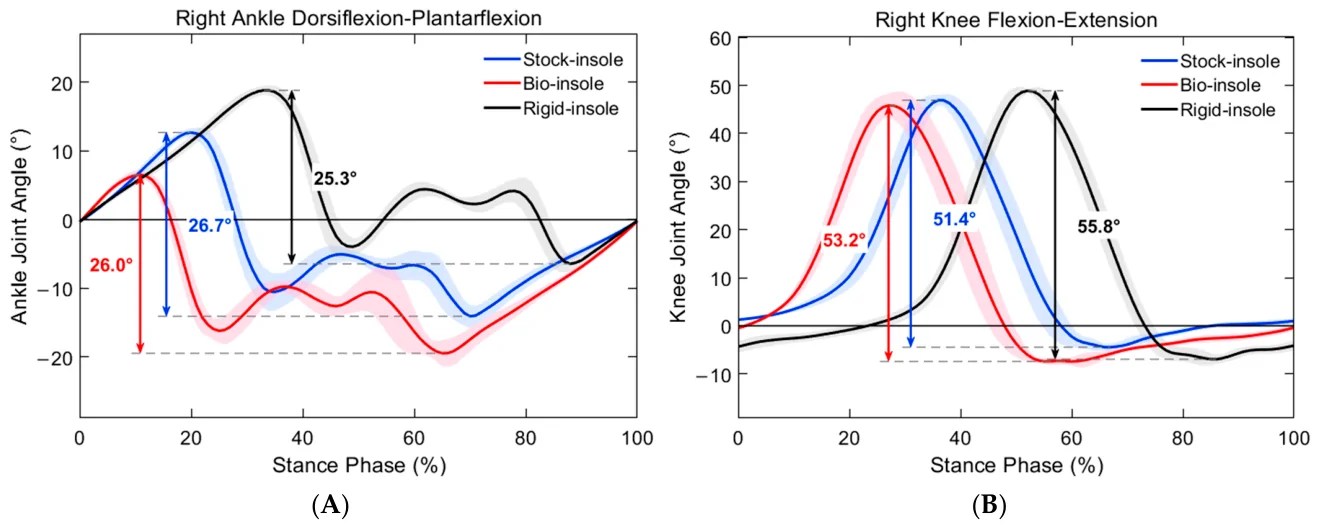
Sagittal-plane joint angle trajectories during 4 m walking under different insole conditions. Time-normalized stance-phase joint angle trajectories are shown for the (**A**) right ankle and (**B**) right knee. Solid lines represent mean trajectories, and shaded regions indicate SD. Vertical arrows and numerical labels indicate sagittal-plane angular excursion for each insole condition, calculated as the difference between the maximum and minimum joint angles during stance.

The average walking speeds during the 4 m walking task were 0.56 m/s, 0.60 m/s, and 0.63 m/s for the stock-insole, bio-insole, and rigid-insole conditions, respectively, showing condition-dependent differences in self-selected walking speed. Figure 7, Figure 8 and Figure 9 summarize the participant-specific wearable outcomes, including regional plantar loading, lower-leg EMG, and sagittal-plane joint kinematics. Percentage and angular-excursion labels are provided only as descriptive visual aids and do not indicate statistical significance.

#### 3.3.1. Plantar Load Redistribution

This wearable assessment examined whether the bio-insole was associated with region-specific plantar load redistribution during walking in this single-participant proof-of-concept evaluation. Regional plantar force was quantified in six predefined plantar regions under the stock-insole, bio-insole, and rigid-insole conditions (Figure 7 and Table 1), and the corresponding peak pressures are summarized in Table 2. Representative plantar pressure maps are provided in Supplementary Figures S1 and S2. Compared with the stock insole, the bio-insole produced a clear redistribution of plantar loading. Regional plantar force increased at the heel, medial arch, and 1st MPJ, from 179.53 ± 20.37 N to 218.51 ± 19.04 N (+21.7%), from 78.85 ± 14.28 N to 105.48 ± 20.26 N (+33.8%), and from 87.68 ± 10.41 N to 138.50 ± 22.55 N (+58.0%), respectively. In contrast, midfoot force decreased from 94.07 ± 13.11 N to 74.95 ± 17.40 N (−20.3%), and lateral arch force showed a modest reduction from 98.19 ± 24.34 N to 90.70 ± 28.78 N (−7.6%). Hallux loading remained minimal in all conditions; however, these near-zero hallux values should be interpreted cautiously because they may partly reflect the limited spatial resolution and low-load sensitivity of the instrumented insole system rather than true absence of loading. Overall, this pattern suggests that the bio-insole shifted plantar load toward the heel, medial arch, and medial forefoot rather than increasing loading uniformly across the foot.

From a biomechanical perspective, the redistribution involved plantar regions with distinct functional roles across stance. Previous studies have reported reduced heel loading in older adults, interpreted as diminished rearfoot engagement or altered balance strategies [17,18,19,20,53,54]. Against this background, the increased heel loading observed here may indicate greater rearfoot engagement during initial contact. The concurrent increase in medial-arch loading, together with reduced midfoot force, may reflect enhanced contact or altered load transfer through the medial support region in this participant with pes cavus. The marked increase in 1st MPJ loading further suggests greater medial forefoot engagement during late stance. Taken together, this region-specific pattern is broadly consistent with the design intention of providing localized support in the heel and arch regions while influencing subsequent forefoot load transfer. However, these interpretations remain indirect, because the present measurements do not resolve the local mechanical contribution of each embedded STG insert.

The peak-pressure results showed a similar pattern. Compared with the stock insole, the bio-insole increased peak pressure at the heel, medial arch, and 1st MPJ from 104.2 ± 20.4 to 186.0 ± 25.9 kPa, from 71.4 ± 11.9 to 142.2 ± 25.4 kPa, and from 107.7 ± 11.7 to 188.3 ± 17.7 kPa, respectively. The agreement between the regional force and peak-pressure findings indicates that the bio-insole redistributed loading toward targeted plantar regions rather than producing a diffuse increase across the foot. However, in this frail older participant with low body mass, sarcopenia, and altered foot morphology, the magnitude of these pressure increases also raises concern regarding localized tissue loading.

Relative to the rigid insole, the bio-insole showed a broadly similar increase in heel and medial-arch loading but a larger increase in 1st MPJ force in this participant. This comparison provides a practical reference indicating that localized STG-based support can modify plantar load transfer in a manner comparable to, but not identical with, a fixed-stiffness insert.

Because this was a descriptive single-participant evaluation, the findings should be interpreted as participant-specific load redistribution rather than evidence of improved or more physiological gait mechanics. Plantar-loading patterns in older adults are influenced by age-related changes in foot structure and walking mechanics [18,19], and elevated plantar pressure has been linked to recurrent tissue breakdown in high-risk diabetic feet, with values around 200 kPa or higher often discussed in the context of high-risk loading or offloading targets [55,56]. However, no universally accepted in-shoe peak-pressure threshold exists for predicting skin breakdown in a frail older foot, and diabetic-foot reference values cannot be directly transferred to the present participant. Moreover, tissue injury risk depends on multiple factors beyond peak pressure alone, including loading duration, cumulative exposure, shear, local tissue tolerance, and underlying clinical condition. Accordingly, the increased heel, medial-arch, and 1st MPJ pressures may reflect functionally relevant load redistribution but also potential tissue-loading risk, which cannot be resolved from this short-duration test. No obvious skin abnormality or clear foot pain was observed during the brief session; however, this does not establish tissue safety or long-term wearability.

#### 3.3.2. Lower-Leg Muscle Activation

The plantar-loading changes observed with the bio-insole were accompanied by differences in lower-leg muscle activation (Figure 8). Compared with the stock insole, the bio-insole was associated with lower measured peak EMG amplitudes in both the TA and GL. Specifically, peak amplitude decreased by 7.8% for the TA and 9.3% for the GL under the bio-insole condition relative to the stock-insole condition. The rigid insole showed a similar reduction in TA peak amplitude (6.9%) but only a small change in GL peak amplitude (1.4%). Thus, in this participant, both non-stock insoles were associated with lower TA activation, whereas the reduction in GL activation was more evident with the bio-insole.

From a functional perspective, this pattern may be relevant to the altered plantar-loading distribution observed under the bio-insole condition. The TA contributes to controlled foot placement and ankle dorsiflexion around initial contact, whereas the GL contributes to plantarflexion and forward progression during stance. In this context, the increased heel loading and greater engagement of the arch/medial forefoot regions under the bio-insole may have modified the mechanical demands placed on these muscles during early and late stance. The lower TA and GL amplitudes therefore suggest that the bio-insole was associated with a modest change in ankle-related muscle demand during the short walking task. The more pronounced reduction in GL amplitude relative to the rigid insole may also indicate that the localized STG-based structure altered late-stance support in a manner that differed from a uniformly rigid insert.

These observations are potentially relevant in the context of aging and sarcopenia, where excessive compensatory lower-leg muscle demand may contribute to fatigue or reduced walking efficiency. However, the functional meaning of the observed EMG reductions remains uncertain. Because EMG amplitudes were not normalized to MVC and only one participant was tested, these findings should be interpreted as descriptive within-participant differences rather than evidence of reduced neuromuscular effort or improved efficiency. Moreover, lower EMG amplitude is not inherently beneficial and could also reflect reduced muscular engagement. Larger controlled studies are required to determine the reproducibility and functional significance of these observed EMG changes.

#### 3.3.3. Sagittal-Plane Lower-Limb Kinematics

Sagittal-plane ankle and knee joint angle trajectories during stance are shown in Figure 9. The stance-phase ankle angular excursions were 26.7°, 26.0°, and 25.3° under the stock-insole, bio-insole, and rigid-insole conditions, respectively, whereas the corresponding knee angular excursions were 51.4°, 53.2°, and 55.8°. Overall, sagittal-plane joint kinematics were broadly similar across the three conditions.

In particular, the bio-insole altered regional plantar loading and lower-leg EMG patterns without producing large observable changes in ankle or knee excursion in this participant. Relative to the stock insole, the ankle excursion under the bio-insole differed by less than 1°, whereas the knee excursion showed only a modest increase. This pattern suggests that the bio-insole was able to influence plantar mechanics without markedly altering the overall sagittal-plane motion pattern of the lower limb during stance. From a design perspective, this pattern is consistent with the intended aim of regional plantar load modulation, namely, to avoid large unintended restrictions or compensatory motions at the ankle or knee during walking.

Comparison with the rigid insole provides additional context. The rigid insole showed a slightly smaller ankle excursion and a larger knee excursion than the stock insole, whereas the bio-insole remained closer to the stock condition. Although these differences were small, this pattern is consistent with the possibility that localized rate-adaptive support may preserve more natural lower-limb motion than a uniformly rigid insert while still modifying plantar load transfer in this participant. In this sense, the kinematic findings complement the plantar loading and EMG results by suggesting that the bio-insole may provide regional mechanical modulation without large observable changes in gross sagittal-plane gait kinematics.

However, these interpretations should remain cautious. The observed angular-excursion differences were small and were derived from an IMU-based motion capture system; they should therefore be interpreted descriptively rather than as definitive evidence of condition-specific changes in joint ROM. Accordingly, the present data support only the preliminary conclusion that no large sagittal-plane kinematic alterations were observed under the bio-insole condition in this single-participant assessment.

### 3.4. Overall Implications and Limitations

Across the material-level and wearable assessments, the findings provide preliminary support for the feasibility of an STG-based passive rate-adaptive insole. The DS30-STG composite exhibited clear rate-dependent stiffening and stable short-term cyclic behavior, while the customized bio-insole was associated with region-specific plantar load redistribution, lower measured TA and GL activation, and broadly preserved sagittal-plane ankle and knee kinematics in the tested participant. Together, these results suggest that embedding STG within a compliant DS30 backbone can provide localized plantar mechanical modulation through a passive material-based strategy inspired by one functional aspect of foot stiffness adaptation during gait.

From a design perspective, the bio-insole was associated with a directional redistribution pattern, with greater heel, medial-arch, and 1st MPJ loading together with reduced midfoot loading. This pattern was accompanied by lower measured lower-leg EMG amplitudes and no large observable changes in gross sagittal-plane joint excursion. These findings provide preliminary support for the concept that a localized rate-adaptive insole may influence plantar load transfer and ankle-related muscle demand without markedly altering overall sagittal-plane lower-limb motion.

However, the wearable evidence remains exploratory. The human evaluation involved only one older adult, and repeated steps represented within-participant observations rather than independent subject-level replicates. No inferential statistics were performed, and no healthy control group, normative plantar-loading target, or clinically validated tissue-safety threshold was available. Accordingly, the observed loading changes should be interpreted as participant-specific load redistribution rather than evidence of improved, more physiological, or clinically beneficial gait mechanics.

The tissue-loading implications also require caution. Although the increased heel, medial-arch, and 1st MPJ loading may be biomechanically relevant to rearfoot contact, arch support, and late-stance forefoot engagement, the corresponding peak-pressure increases may also indicate undesirable localized tissue stress in a frail older foot with reduced muscular and soft-tissue reserve. Importantly, these pressure-redistribution findings were derived from only three trials of a 4 m walking task in a single session. Therefore, the tissue-loading safety of the observed plantar pressure pattern cannot be judged from this short exposure alone, nor can it support any recommendation for prolonged or real-world use. Safety observations were limited to immediate post-session visual inspection and participant self-report, and no follow-up skin check was performed several hours after testing or on the following day. Delayed adverse skin responses and the safety of prolonged or repeated use therefore remain unknown.

Additional methodological limitations should also be noted. Walking speed was self-selected rather than controlled, and the slightly higher mean speeds in the bio-insole and rigid-insole conditions may have confounded plantar loading, EMG, and kinematic outcomes. Only one shoe type was tested, and the wearable assessment was limited to short straight-line walking. Formal patient-reported outcomes, cumulative loading metrics, long-term safety monitoring, and measures of balance, postural stability, perceived stability, confidence, and fall risk were not included. Therefore, although fall risk was part of the broader clinical motivation for studying sarcopenic older adults, the present experiment does not provide evidence regarding stability improvement, confidence change, or fall-risk reduction. In addition, EMG amplitudes were not normalized to MVC, limiting interpretation to within-participant, within-muscle comparisons, and low regional values, particularly in the hallux region, may have been affected by the sensitivity and spatial resolution of the instrumented insole system, given the limited number of sensels representing small anatomical regions in the 20 × 12 pressure grid.

The material-level interpretation also has limitations. A DS30-only wearable control was not included in the human testing; therefore, the in-shoe contribution of STG cannot be fully separated from that of the DS30 backbone geometry. The cyclic compression test addressed only short-term repeatability, and the mechanical characterization was performed using cylindrical sandwich specimens rather than final insole-shaped structures. In addition, the repeated mechanical tests were technical repeats rather than independent specimen replicates, and batch-to-batch variability of the hand-fabricated DS30-STG composite was not evaluated. Furthermore, the local deformation rate and real-time stiffening state of the STG during walking were not directly measured, so the timing and magnitude of in-shoe STG activation remain unknown. Future studies should therefore include larger and more diverse cohorts, standardized gait conditions, appropriate wearable controls, longer-duration wear, direct measurements of regional insole deformation, independently fabricated specimens from multiple batches, and longer-term assessment of durability, comfort, tissue-loading safety, and functional outcomes.

## 4. Conclusions

This study developed a bioinspired passive rate-adaptive insole by embedding STG within a DS30 elastomeric backbone. Mechanical testing demonstrated clear compression-rate-dependent stiffening of the DS30-STG composite, with apparent modulus increasing as compression speed increased. Cyclic compression over 100 cycles at 40% strain further showed stable short-term repeatability under the tested laboratory conditions, although these tests did not establish long-term fatigue durability, wear resistance, or performance during prolonged daily use.

A customized bio-insole was then fabricated for an older adult with sarcopenia, pes cavus, and hindfoot varus tendency and evaluated against stock and rigid insoles during walking. Relative to the stock insole, the bio-insole was associated with higher plantar force and peak pressure at the heel, medial arch, and 1st MPJ, together with reduced midfoot loading, indicating region-specific plantar load redistribution. It was also associated with lower measured peak EMG linear-envelope amplitude of the TA and GL, without large observable changes in sagittal-plane ankle or knee kinematics.

Overall, these findings provide preliminary proof-of-concept support for an STG-based bio-insole as a passive, localized, and rate-adaptive strategy for modulating plantar load transfer while preserving gross lower-limb sagittal-plane motion during short walking. However, because the wearable evaluation was limited to a single participant with a specific clinical profile, the findings should be interpreted as participant-specific rather than generalizable. Future studies should validate this concept in larger and more diverse cohorts, incorporate appropriate wearable control conditions, and assess long-term durability, in-shoe mechanical behavior, individualized STG placement, real-world walking performance, and user-relevant functional outcomes before broader claims regarding clinical utility can be made.

## Figures and Tables

**Table 1:** Phase-window-specific regional plantar forces (N) during 4 m walking under different insole conditions. Data are presented as mean ± SD.

	Stock-Insole	Bio-Insole	Rigid-Insole
Heel	179.53 ± 20.37	218.51 ± 19.04	214.85 ± 21.78
Midfoot	94.07 ± 13.11	74.95 ± 17.40	56.11 ± 9.76
Medial arch	78.85 ± 14.28	105.48 ± 20.26	106.04 ± 19.47
Lateral arch	98.19 ± 24.34	90.70 ± 28.78	90.96 ± 15.33
1st MPJ	87.68 ± 10.41	138.50 ± 22.55	115.99 ± 15.95
Hallux	0.00 ± 0.00	0.23 ± 0.26	0.00 ± 0.00

**Table 2:** Phase-window-specific regional peak pressures (kPa) during 4 m walking under different insole conditions. Data are presented as mean ± SD.

	Stock-Insole	Bio-Insole	Rigid-Insole
Heel	104.2 ± 20.4	186.0 ± 25.9	212.9 ± 37.0
Midfoot	67.5 ± 8.9	62.3 ± 14.4	53.1 ± 10.3
Medial arch	71.4 ± 11.9	142.2 ± 25.4	131.0 ± 18.2
Lateral arch	57.9 ± 17.7	62.5 ± 12.8	78.1 ± 7.5
1st MPJ	107.7 ± 11.7	188.3 ± 17.7	189.9 ± 26.1
Hallux	0.0 ± 0.0	2.0 ± 2.3	0.0 ± 0.0

## Data Availability

The de-identified data supporting the findings of this study, including specimen-level mechanical data and participant-level processed walking outcomes, are available from the corresponding author upon reasonable request, subject to the conditions of the ethics approval and participant consent.

## Supplementary Materials

The following supporting information can be downloaded at https://www.mdpi.com/article/10.3390/biomimetics11100699/s1. Figure S1: Plantar pressure distribution patterns during 4 m walking under different insole conditions; Figure S2: Plantar pressure distributions across stance subphases under different insole conditions; Figure S3: Wearable sensor setup for biomechanical and plantar pressure assessment; Figure S4: Foot-segment velocity assessment during locomotion at different speeds; Table S1: Apparent compressive modulus (kPa) values of DS30, DS30-STG, and EC50-STG specimens at different compression speeds.

## Abbreviations

The following abbreviations are used in this manuscript:

STG	Shear-stiffening gel
PBDMS	Polyborondimethylsiloxane
EMG	Electromyography
RA-SLS	Rate-activated standard linear solid
TA	Tibialis anterior
GL	Lateral gastrocnemius
MVC	Maximum voluntary contraction
1st MPJ	First metatarsophalangeal joint
ROM	Range of motion
SD	Standard deviation
