# Supplementary material for: A Bioinspired Insole Based on Shear-Stiffening Gel for Plantar Load Modulation: Material Characterization and a Single-Case Wearable Evaluation
Source: Biomimetics (Basel). 2026 Oct 2;11(10):699. doi: 10.3390/biomimetics11100699 (PMC13702761; doi:10.3390/biomimetics11100699)
Supplement: Supplementary file 1 [file biomimetics-11-00699-s001.zip › biomimetics-4572596-supplementary.pdf]

Supplementary Materials

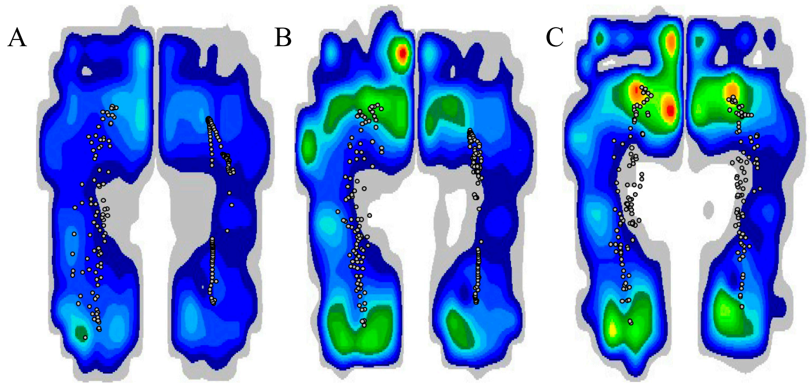

Supplementary Figure S1. Plantar pressure distribution patterns during 4 m walking under different insole conditions.

Average plantar pressure distribution patterns obtained during the 4-m walking test with (A) the stock insole, (B) the bio-insole, and (C) the rigid insole. Warmer colors indicate higher plantar pressure, whereas cooler colors indicate lower plantar pressure.

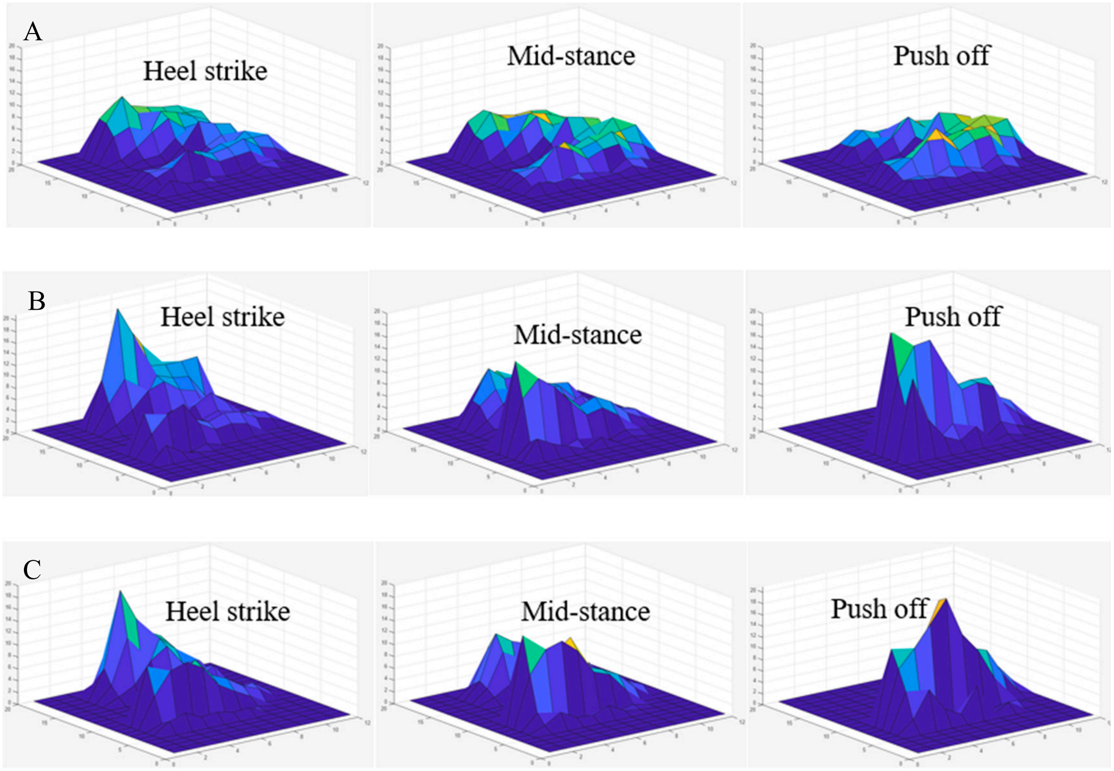

Supplementary Figure S2. Plantar pressure distributions across stance subphases under different insole conditions.

Three-dimensional plantar pressure distributions obtained during heel strike, mid-stance, and push-off under (A) the stock-insole, (B) the bio-insole, and (C) the rigid-insole conditions. Higher peaks represent greater localized plantar pressure.

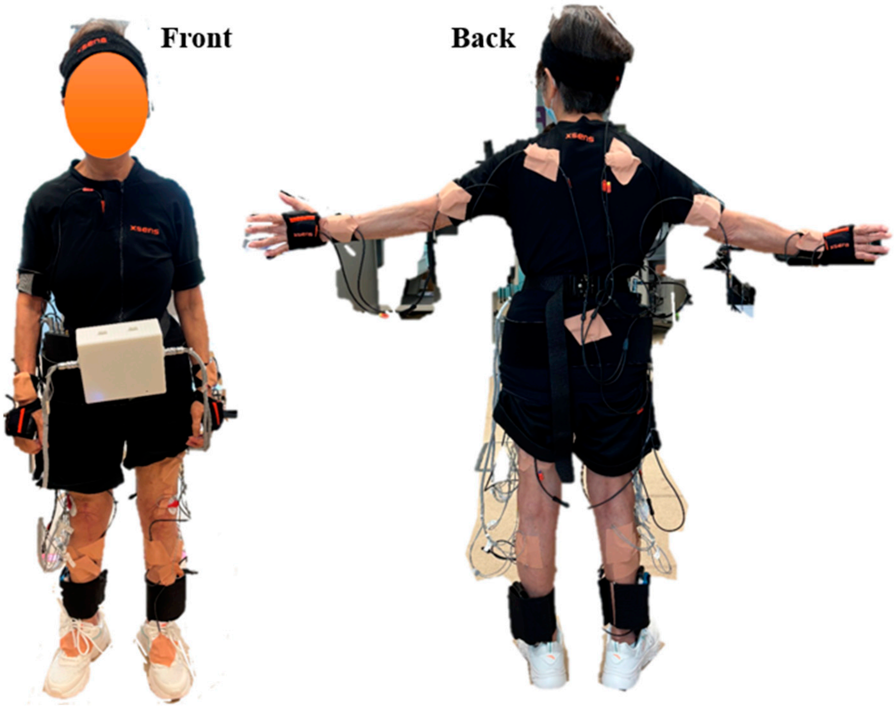

Supplementary Figure S3. Wearable sensor setup for biomechanical and plantar pressure assessment.

Photographs showing the front and back views of participant B037 wearing the integrated wearable measurement system, including surface EMG electrodes and recording device, an IMU-based Xsens motion capture system, and Noraxon medilogic WLAN pressure insoles.

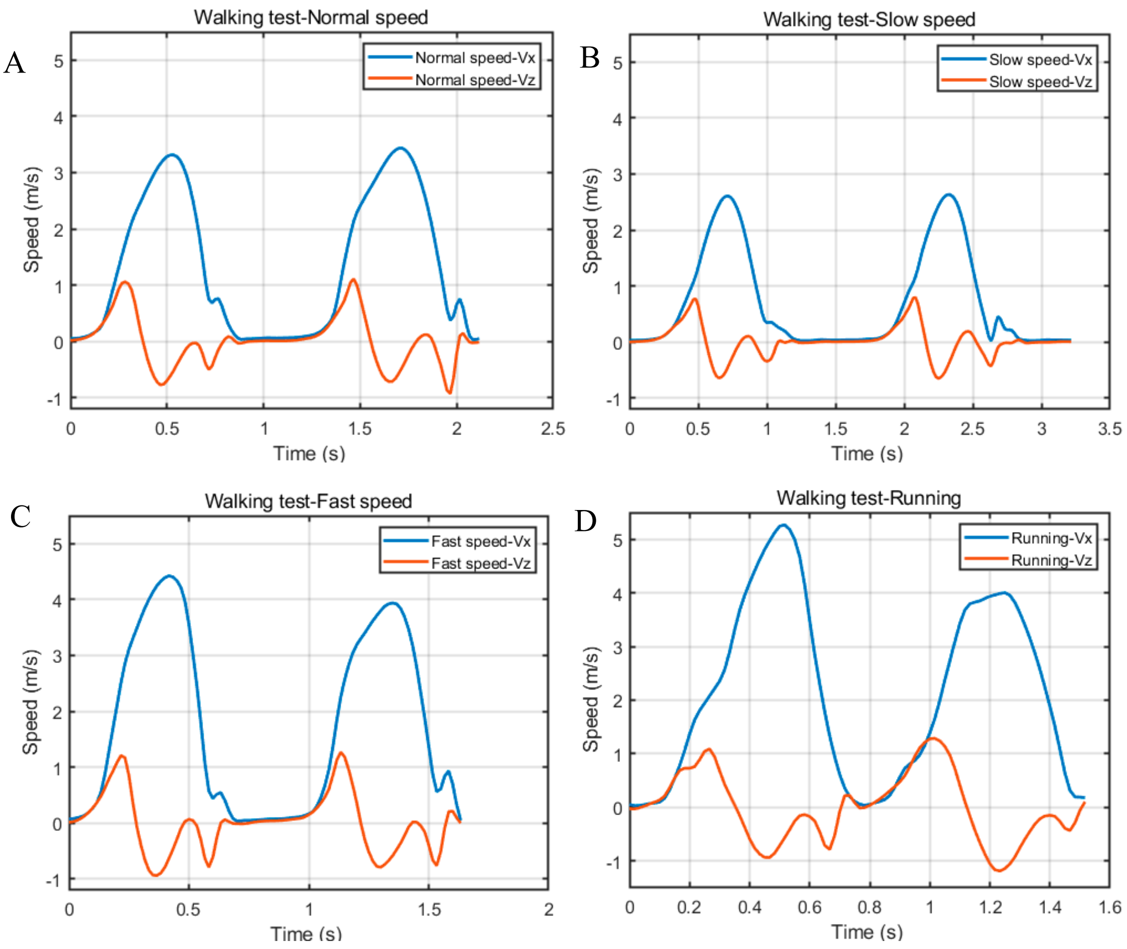

Supplementary Figure S4. Foot-segment velocity assessment during locomotion at different speeds.

The horizontal velocity component, Vx blue solid line, and vertical velocity component, Vz orange solid line, of the foot segment were measured using the Xsens system during (A) normal walking, (B) slow walking, (C) fast walking, and (D) jogging. The vertical velocity component showed transient peaks and rapid changes around repeated foot landing/loading events. These velocity transients provide preliminary gait-related context for the rate-dependent insole design and suggest that walking involves rapid foot-motion events during gait phases in which the heel and medial arch STG inserts were expected to experience increased loading. However, because foot-segment velocity is not equivalent to local insole deformation, these data should not be interpreted as direct measurements of the compression or shear deformation rate of the embedded STG inserts.

Supplementary Table S1. Apparent compressive modulus (kPa) values of DS30, DS30-STG, and EC50-STG specimens at different compression speeds.

Values are presented as mean ± SD.

Sample	**30 mm/min**	**300 mm/min**	**1000 mm/min**	**2000 mm/min**
EC50-STG	146.0 ± 0.8	271.1 ± 7.8	416.6 ± 9.1	616.8 ± 2.1
DS30-STG	262.1 ± 17.0	401.7 ± 1.3	485.7 ± 3.5	553.0 ± 1.1
DS30	683.2 ± 20.6	726.3 ± 11.2	797.1 ± 10.3	734.8 ± 47.2
